# Black Soldier Fly Promoted Bioconversion of Tomato Toxic Plant Biomass to Safe, Functional Animal Feed

**DOI:** 10.3390/molecules31071098

**Published:** 2026-03-27

**Authors:** Dionysios T. Pavlopoulos, Evgenia-Anna Papadopoulou, Konstantinos M. Kasiotis, Serkos A. Haroutounian

**Affiliations:** 1 Laboratory of Nutritional Physiology & Feeding, Department of Animal Science, Agricultural University of Athens, Iera Odos 75, 11855 Athens, Greece; dion.pavlo@aua.gr; 2 Laboratory of Pesticides’ Toxicology, Scientific Directorate of Pesticides’ Control & Phytopharmacy, Benaki Phytopathological Institute, Stefanou Delta 8, 14561 Athens, Greece; a.papadopoulou@bpi.gr (E.-A.P.); k.kasiotis@bpi.gr (K.M.K.)

**Keywords:** tomato plant biomass, *Solanum lycopersicum*, insect meal, BSF rearing, plant biomass valorization, solanaceae cultivation byproducts, circular economy

## Abstract

The escalating demand for sustainable, nutrient-dense feeds underscores the need to valorize the agro-industrial byproducts utilizing innovative bioconversion strategies. In this context, we have studied the feasibility of incorporating tomato (*Solanum lycopersicum*) cultivation residues into Black Soldier Fly (BSF) larvae diets to produce high-protein insect meals. These residues are known to contain the naturally occurring toxic steroidal alkaloids tomatidine and α-tomatine, prohibiting their incorporation into human and animal diets. Herein, the tomato cultivation biomass was dried and mill-ground, and its varying volumes were incorporated into standard poultry feed (seven diet levels with 0–100% biomass inclusion) and tested in BSF-larvae-rearing trials to produce insect meals. The optimal results with respect to larvae growth, protein accumulation (highest value = 30.61%), lipid–fiber content, and antioxidant capacity were determined for insect meals obtained from BSF larvae reared with a ration composed of 40% tomato plant biomass. In addition, the toxicity of this insect meal was substantially low, as a consequence of the observed groundbreaking reduction in the contained toxic steroidal alkaloids α-tomatine and its aglycone tomatidine. The results herein reveal the efficacy of the BSF-larvae-rearing process in acting as a biological filter for the bioconversion of the toxic tomato cultivation waste into a functional, safe, and protein-rich livestock feed, supporting the principles of a circular economy.

## 1. Introduction

The continuous growth of the global population has initiated an increasing demand for larger quantities of sustainable nutrient-dense foods and feed ingredients. This exerts an enormous pressure on agricultural systems and natural resources according to the 2021 report of the United Nations Department of Economic and Social Affairs [1], leading to the degradation of environment and soils [2]. The latter are included among the main causes of climate change that has recently been affecting humanity, and herein emerges the necessity to introduce circular economy principles into the food production procedures and processes, such as the development and utilization of waste-management infrastructures [3]. In this regard, the upscaling of agro-industrial wastes to high-nutritional-value animal feed through their bioconversion by insect rearing constitutes an intriguing subject [4], capable of contributing greatly to environmental protection and food-system resilience [5].

Among various insect species investigated to date, the Black Soldier Fly (BSF, *Hermetia illucens*) is included among the most efficient bio-converters. It is well-established that it displays the unique ability to thrive on a wide range of organic substrates, which are rapidly transformed into protein-rich meals suitable for livestock feeding [6]. Thus, the BSF have been included by the European Union amongst the officially approved species that exhibit the capability to produce insect meals allowed for the incorporation into livestock-rearing diets [7]. Consequently, a vigorous research effort has been initiated towards the exploitation of a broad variety of agricultural cultivations, and their processing byproducts, such as plant residues, agro-industrial wastes, and animal manure as BSF-rearing feeds [8].

In the context of our ongoing research interest concerning the circular economy advancement and focusing on the valorization potentials of a broad variety of cultivation and agro-industrial byproducts and wastes [9,10,11], we were intrigued to investigate the incorporation of the tomato plant cultivation biomass into BSF-larvae-rearing rations. Tomato fruit is harvested from *Solanum lycopersicum*, a Solanaceae family plant that comprises the most consumed globally fruit, with an annual consumption volume reaching 200 million tonnes [12]. It must be noted, however, that, although tomato fruit and its processing byproducts have been extensively investigated and valorized by the food, pharmaceutical. and cosmetic industries, the aerial parts of the tomato plant that are produced in large amounts—consisting of leaves, stems, and axillary shoots—are less studied and utilized. The main drawback of harnessing this biomass rich in high-nutritional-value proteins, minerals, fiber, and antioxidant phytochemicals [13] is the concomitant presence of toxic alkaloids. Thus, the green parts and shoots of the tomato plant are known to contain large amounts of tomatidine, a naturally occurring steroidal alkaloid, and its glycoalkaloid metabolite α-tomatine, which constitute a serious obstacle for tomato biomass utilization in human and animal diets [14]. Their consumption can cause gastrointestinal and neurological disorders (nausea, diarrhea, vomiting, and headache) or even death in large quantities [15]. Thus, the management of these residues represents a major challenge, particularly for the intensive horticultural systems where the large quantities of biomass accumulated on-site alter the microbial fauna, contributing to the greenhouse gas emissions and pathogens’ propagation [16].

Therefore, the successful integration of tomato plant biomass into BSF larvae diets is expected to reduce the environmental impacts associated with the accumulation of crop-residues and simultaneously provide an alternative protein source. As global projections estimate that, over the coming decades, humanity will face a significant rise in feed demand and protein scarcity, the exploitation of plant residues as functional substrates for insect meal production represents an important strategic direction that contributes to the mitigation of the growing competition between human and animal nutrition [17,18,19].

Recent studies on BSF larvae rearing with a broad variety of cultivation and agro-industrial byproducts have provided promising results with respect to larval growth, nutrient assimilation, and the enrichment of insect meals with bioactive compounds [20]. However, research endeavors using as the primary substrate the toxic aerial parts of tomato plants remain scarce [21], despite their abundance and year-round availability from greenhouse production systems, presumably because their compositional attribute may potentially influence the larvae metabolism [22].

The present endeavor aspires to investigate the feasibility of incorporating tomato plant residues into BSF larvae diets to produce non-toxic, functional insect meals. For this purpose, the outcome of BSF insect rearing incorporating various proportions of the tomato plant biomass into their diet is evaluated through the assessment of the growth performance of the reared larvae and the nutritional value and toxicity of the produced insect meal. In addition, the phenolic content and antioxidant capacity of the produced insect meals were also determined, aiming to evaluate the potential changes in their functional and nutritional quality. The results herein are expected to contribute towards the development of a novel, environmentally responsible bio-conversion strategy for the sustainable production of animal feed. For this purpose, the valorization of tomato cultivation toxic residues into insect farming is expected to create a pivotal link between the reduction in agricultural waste and the fulfillment of future nutritional demands.

Finally, it must be noted that this study addresses a critical knowledge gap regarding whether toxic tomato cultivation residues can be safely valorized, demonstrating, for the first time, that a hazardous agro-industrial waste can be bio-converted by BSF larvae, providing a safe and sustainable insect meal applicable in animal nutrition.

## 2. Results and Discussion

### 2.1. Larvae Growth

The inclusion of tomato biomass in the BSF larvae diet had different effects on their growth parameters, depending on the incorporated amount of tomato, as shown in Table 1. The recorded weight values are comparable to those determined for the control diet (containing 0% of tomato biomass). Specifically, the addition of 10% tomato biomass did not exhibit a statistical difference as compared to control, while the 20%, 40%, 60%, and 80% diet groups displayed an informal statistical grouping. On the contrary, a different outcome was determined for larvae reared with 100% tomato biomass, since a significant reduction in their weight was observed.

It must be noted that, for treatments containing 10–60% tomato plant biomass, the larvae body weights were ranged between 193.9 and 235.8 mg, while their body lengths remained within a relatively narrow interval (21.1–24.0 mm), indicating that partial substitution did not induce pronounced growth impairment. Within this intermediate range, larvae reared with diets containing 40–60% tomato biomass displayed balanced growth metrics, with body weights and lengths remaining within the variability observed for the control group.

In the terms of the larvae length, the respective results revealed as less effective the utilization of 100% tomato plant biomass. The 80% biomass incorporation resulted in a reduced larvae length and an average body weight growth. On the other hand, the incorporation of 40%, and 60% biomass displayed the best performance with respect to the length of the larvae, comparable to the respective values observed for the control sample (0% inclusion). Finally, the larvae fed with 10% and 20% plant biomass displayed the best performance with respect to their weight and length.

**Figure 1:**
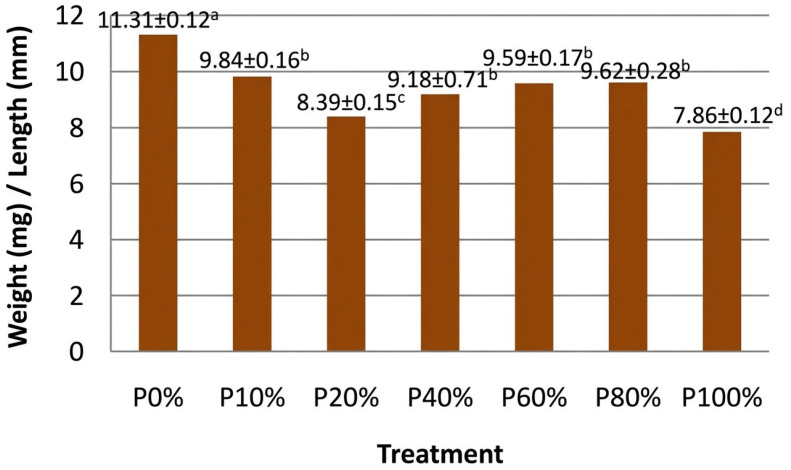
Comparative analysis of the weight-to-length ratio of BSF *larvae* for different dietary treatments [P = (Plant Biomass)]. Exponents a, b, c, and d indicate the respective grouping (a < b < c < d).

As illustrated in Figure 1, the calculated rate of weight-to-length growth for the control treatment (P0%) presented the highest value, while the lowest was observed for the 100% tomato biomass treatment (P100%). Intermediate inclusion levels (P10, P40–P80%) showed relatively high values, supporting the ability of BSF larvae to maintain a satisfactory growth morphology under partial substitution conditions. On the other hand, the weight-to-length ratio for the intermediate inclusion levels (10, 40, 60, and 80%) displayed a similar behavior, with weight/length values ranging from 9.18 to 9.84, suggesting the maintenance of larvae welfare and a favorable growth morphology, and indicating an adequate digestibility of the substrate [23]. It must also be noted that, although the control diet exhibited the highest ratio (11.31), the moderate inclusion levels did not lead to a sharp decline, indicating the potential of feeding the BSF larvae efficiently with mixed substrates combining conventional feed with tomato plant biomass.

With respect to these observations, the significantly lower weight-to-length ratio (7.86) determined for the larvae reared with 100% tomato biomass, as a consequence of their reduced body weight (116.6 mg) and length (14.8 mm), may be attributed to various limitations associated with the high content of the biomass in the structural fibers and other less digestible components. The latter are capable of constraining the nutrients’ assimilation and energy allocation for somatic growth. Similar constraints have been reported in previous studies investigating the high inclusion levels of plant-derived substrates in BSF diets [22,24]. The slightly reduced performance observed for the 20% inclusion level may also reflect a transitional dietary effect, since, at this substitution rate, the partial replacement of the control diet alters the protein-to-energy balance and increases the fiber content, affecting the nutrient availability. Such non-linear responses to gradual dietary changes have also been previously reported for BSF larvae and have been attributed to threshold effects in their nutrients’ dilution and digestibility [25,26].

### 2.2. Nutritional Aspects of Insect Meals, Tomato Plant Biomass, and Poultry Feed

The nutritional composition of BSF-derived insect meals was strongly influenced by the incorporated proportion of tomato biomass into their diet, confirming the substrate-dependent plasticity of insect body composition [27,28,29].

To better contextualize the nutritional composition of the produced insect meals, Table 2 illustrates the outcome of the corresponding analyses for the tomato plant biomass and standard poultry feed used in the study. These reference values highlight the intrinsic differences between the substrates and the resulting BSF-derived meals, demonstrating clearly that the observed compositional shifts in Table 3 are primarily driven by the BSF bioconversion.

#### 2.2.1. Protein Content

The protein concentration of the produced insect meals exhibited a strong dependance on the utilized tomato biomass volume (*p* < 0.001). In particular, the protein values were progressively increased from 20.58% for the control treatment (T0%) to a maximum of 30.61% for a 40% inclusion level (T40%). The latter represents the highest protein content among all studied treatment levels, significantly exceeding both the control and the highest substitution levels (T100%). Beyond the 40% inclusion level, the protein concentration displayed a decline to 29.04% for T60% and 29.72% for T80%, followed by a pronounced decrease for 100% inclusion (18.39%), which corresponded to the lowest recorded value.

The protein content peak observed for the insect meal obtained from 40% tomato biomass inclusion suggests that the moderate incorporation of tomato biomass promotes protein deposition, presumably because of an improved nutrient balance and complementary substrate effects. On the other hand, the incorporation of excessive amounts of biomass reduces protein accumulation, possibly due to the increased fiber content and reduced nutrient digestibility. Similar non-linear responses have been previously reported for BSF reared with plant-based byproducts, indicating that the utilization of intermediate fiber levels optimizes the protein deposition before nutritional imbalances constrain the assimilation efficiency [24,29]. Finally, the relatively high standard deviation observed for 100% inclusion is indicative of nutrient instability under extreme dietary conditions.

#### 2.2.2. Fiber Fractions

The crude fiber content, in combination with the presence of its structural components, lignin and cellulose, is widely recognized the limiting factor for nutrients’ digestibility and energy utilization, particularly for monogastric feeding systems [30]. Apart from the minor fluctuations observed for intermediate levels, the overall trend indicates a greater structural carbohydrate accumulation with increased tomato biomass utilization. In particular, the crude fiber content was progressively increased with respect to the included tomato biomass (*p* < 0.001), displaying values ranging from 6.45% for the control and 40% treatments to 11.87% for feeding with 100% tomato biomass. Similar trends were revealed for lignin and cellulose fractions, with the highest values consistently recorded for diets dominated by large amounts of tomato biomass.

A similar increasing pattern was observed for the lignin content (*p* < 0.01), which increased from 1.82% (T0%) to 3.29% (T100%), and for cellulose (*p* < 0.05), which exhibited the highest values of 15.74 and 15.31% for 20 and 40% biomass inclusion, respectively.

Overall, the best values for the crude fiber (6.45%) and lignin (2.47%) contents were determined for the 40% tomato biomass inclusion level. These values are considerably lower as compared to those determined for higher inclusion levels. This is a nutritionally important finding since excessive lignocellulosic fractions may impair nutrient digestibility, reduce metabolizable energy availability, and negatively affect feed efficiency [31].

Thus, the intermediate biomass inclusion levels appear to allow for the partial utilization of plant structural components without exceeding thresholds that compromise the nutritional functionality of the resulting insect meal.

#### 2.2.3. Lipid and Ash Content

The ash content was significantly influenced by the tomato biomass inclusion (*p* < 0.001). While the ash values remained relatively stable between T0% and T40% (10.62–11.05%), a marked increase was observed for higher substitution levels, reaching 23.18% for 100% inclusion. The observed moderate ash level for 40% inclusion suggests a balanced mineral contribution without excessive accumulation that could negatively affect the feed formulation flexibility or palatability [32].

On the other hand, the total lipid content was also significantly affected (*p* < 0.001), since the highest lipid concentration was recorded for the control and the low-to-moderate inclusion levels (approximately 29–31%), with T10% showing the maximum value (30.59%). However, beyond 40% inclusion, the lipid content progressively declined, dropping sharply for 80 and 100% biomass inclusion (18.61 and 6.62%, respectively). This pronounced reduction is consistent with previous findings, indicating that substrates characterized by high fiber and low lipid availability limit the larvae fat deposition [25]. The reduced lipid accumulation under a high tomato biomass substitution may reflect the metabolic prioritization of maintenance over storage processes, thereby decreasing the energetic value and processing flexibility of the resulting insect meal.

### 2.3. Phenolic Content and Antioxidant Activity

The antioxidant capacity of BSF insect meals was clearly affected by the incorporated amount of tomato plant biomass in their diets (Table 4). Moderate amounts enhanced both the phenolic content and antioxidant activity, while the incorporation of larger amounts resulted in a marked decline in antioxidant capacity.

Specifically, the phenolic content (*p* < 0.001) was increased in analogy with the tomato biomass incorporation, reaching the highest values for the 20% and 40% levels (respectively, 19.83 and 19.96 mg/mL). This trend suggests an improved availability or partial transfer of tomato-derived phenolic compounds for the intermediate inclusion levels [33]. In contrast, a further increase in the incorporated amounts of tomato biomass resulted in a progressive decrease in phenolic content (11.47 and 8.74 mg/mL) for 80 and 100% inclusion, respectively, the latter representing the lowest recorded value. This decrease indicates a limited assimilation efficiency and/or reduced metabolic incorporation of bioactive compounds under high substitution levels.

The determination of the DPPH^•^-radical-scavenging capacity (*p* < 0.001) followed a similar pattern. Treatments containing 10–40% tomato biomass exhibited higher antioxidant activity, with the maximum value (27.11 mg/mL) observed for 10%. For higher inclusion levels, the determined antioxidant capacity declined sharply with the lowest value observed for the incorporation of 100%, indicating that the inclusion of high tomato byproduct levels may be associated with an increased fiber content and reduced metabolic efficiency [34].

In contrast, the corresponding FRAP values were not significantly affected by dietary treatments (*p* > 0.05). Although the numerically higher reducing capacity was observed for 40% biomass inclusion (0.69 mg/mL), the differences among treatments were not statistically significant. This suggests that, while the radical scavenging capacity (DPPH^•^) is highly responsive to the substrate composition, the ferric reducing ability appears more stable across dietary treatments.

Overall, the results indicate that moderate tomato biomass inclusion levels (20–40%) optimize the antioxidant profile of BSF-derived meals by enhancing the phenolic content and radical scavenging activity. Conversely, excessive substitution negatively affects the antioxidant yield and functional capacity. These findings support the use of balanced substrate formulations to improve the functional and nutraceutical quality of insect-derived feed ingredients [34,35].

### 2.4. Presence of Toxic Alkaloids

The BSF-*larvae*-feeding bioconversion substantially reduced the concentrations of toxic steroidal alkaloids, α-tomatine and its aglycone tomatidine, contained in tomato plant biomass (Table 5). Specifically, the recorded initial levels of these compounds were particularly high for the vegetative tissues of tomato plants (α-tomatine 1166.9 ppm and tomatidine 70.6 ppm), where these compounds play a defensive role against herbivores and pathogens [14,36]. As is expected, in the control feed (0% tomato biomass inclusion), both α-tomatine and tomatidine levels were below the limit of quantification (<LOQ), confirming that these toxic alkaloids were not endogenously present in the BSF and/or poultry feed.

On the contrary, the α-tomatine and tomatidine concentrations were considerably reduced (respectively, by 93% and 40%) in insect meal produced from BSF larvae reared with the feed determined herein as optimal (containing 40% of tomato plant biomass). Finally, it is noticeable that the insect meal obtained by BSF larvae fed with 100% tomato plant biomass was also determined to contain relatively low amounts of α-tomatine and tomatidine (respectively, 216.8 and 34.6 ppm), indicating the bioconversion efficacy of BSF insects. To provide a useful toxicological insight, the literature-reported LD_50_ values for the oral consumption of animals for α-tomatine is 500 ppm (500 mg/kg body weight, bw) [14] and for tomatidine is 833 ppm [37]. The LD_50_ value is a substantial indicator of acute exposure effects, interacting with various aspects of this study (e.g., the first administration of the feed). More specifically, based on the α-tomatine concentration in the insect meal obtained by BSF rearing with 40% tomato biomass (109.6 mg/kg), considering the 0.25 kg body weight of an animal (e.g., newborn chicken) and an average daily consumption of approximately 0.125 kg of the meal (with an admission of the comparability of the data between rats and chicken), the derived daily consumption is 54.8 mg/kg bw, a much lower value than the LD_50_. In addition, it is apparent that as body weight increases, the risk further decreases. For chronic effects, to our knowledge there are no established chronic safety reference endpoints. More specifically, the European Food Safety Authority (EFSA) found insufficient repeated-dose and chronic data to derive a chronic safety reference point and specifically noted a lack of sufficient toxicokinetic and repeated-dose studies for tomato glycoalkaloids [38].

These results are indicative of BSF’s capability to limit the transfer of tomato biomass toxic alkaloids into the larvae biomass that constitutes the produced insect meal, presumably through a combination of enzymatic degradation, metabolic transformation, and selective excretion processes. In this regard, previous studies have indicated that glycosylated steroidal alkaloids such as α-tomatine are particularly susceptible to hydrolysis, yielding the aglycone tomatidine as an intermediate or degradation product [35]. Moreover, the low accumulation of tomatidine in the BSF-derived insect meals is also indicative of a strongly constrained downstream retention for both alkaloids, even when large amounts of tomato biomass are used [39]. Differences in the chemical structures, polarities, and metabolic stability between α-tomatine and tomatidine rationalize their differential persistence, with the aglycone form generally exhibiting a higher resistance to complete degradation. Nevertheless, the concentrations detected for the BSF-derived insect meals are markedly reduced as compared to the plant biomass substrate.

From the agro-industrial valorization perspective, the significant attenuation of tomato alkaloids through BSF bioconversion is particularly relevant, since these molecules are commonly regarded as toxic antinutritional compounds with limited acceptable concentrations in feed-related applications. Their substantial reduction through this insect-mediated processing enhances the suitability of tomato plant biomass residues for the integration into circular bioconversion chains and simultaneously improves the compositional profile of the resulting insect-derived biomass.

## 3. Materials and Methods

### 3.1. Chemicals and Feed

Analytical-grade methanol (Fisher Chemicals, Hampton, NH, USA) was used for sample extractions which were utilized for the antioxidant capacity evaluation. Folin–Ciocalteu reagent, 2,2-diphenyl-1-picrylhydrazyl (DPPH), and 2,4,6-tris(2-pyridyl)-s-triazine (TPTZ) were purchased from Sigma-Aldrich (Burlington, MA, USA). Trolox and vanillin were obtained from Acros Organics (Geel, Belgium). Anhydrous sodium carbonate, sulfuric acid (98%), and hydrochloric acid were supplied by Chem-Lab, while glacial acetic acid was obtained from Sigma-Aldrich (Burlington, MA, USA). Aluminum chloride hexahydrate was purchased from Fluka and sodium acetate hydrate from Merck (Darmstadt, Germany). Ferric chloride hexahydrate and iron sulfate heptahydrate were supplied by Alfa Aesar (Heysham, UK). 3,5-Dinitrosalicylic acid, potassium sodium tartrate, crystalline phenol, and cetyltrimethylammonium bromide were obtained from Panreac AppliChem (Darmstadt, Germany). Sodium hydroxide and potassium hydroxide were purchased from Penta Chemicals. Boron trifluoride (14% in methanol) was supplied by Sigma-Aldrich (Burlington, MA, USA).

The studied insect meals were extracted with LC/MS-grade methanol (≥99.99%, Fisher). The following solvents were used for the UPLC-MS determinations: methanol (hypergrade for LC-MS, LiChrosolv, Supelco, Bellefonte, PA, USA), water (LC-MS grade, LiChrosolv, Supelco), formic acid (98–100%, LiChropur, Sigma-Aldrich), and ammonium formate (99+%, Chem-Lab NV, Zedelgem, Belgium). The analytical standards used for the identification and quantification of alkaloids were tomatidine (10 mg, Extrasynthese, Genay, France) and α-tomatine (≥95%, 100 mg, Extrasynthese, Genay, France)

The poultry feed used in this study was Fyrko EL 1500002 pellets, commercially available grains for farmyard chickens’ growth, purchased from Fyrko SA (Corinth, Greece).

### 3.2. Isolation of Tomato Plant Cultivation Biomass

Tomato plants (*Solanum lycopersicum*) were cultivated under standard horticultural practices throughout the completion of their fruit-harvesting cycle. After harvesting, the remaining aerial biomass (stems, leaves, and non-commercial fruit residues) was collected from the field and transported to the laboratory for further processing. This material was initially spread in a shaded, well-ventilated area to facilitate their dehydration and prevent the photodegradation of contained phytochemicals. Once pre-dried, the biomass was transferred to a controlled drying chamber and for complete dehydration to ensure long-term stability and prevent microbial growth. Dried plant tissues were subsequently mill-ground to provide a fine, homogeneous powder which was served as the vegetal substrate for the dietary treatments applied in the terms of BSF-larvae-rearing experiments.

### 3.3. BSF Feed Preparation

**Figure 2:**
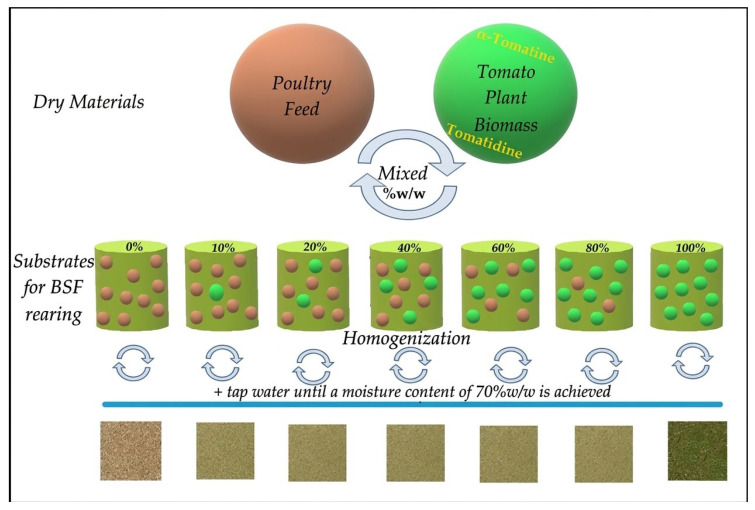
BSF diets preparation procedure from poultry feed and tomato plant biomass.

The experimental BSF diets were formulated by combining a standard poultry feed (also used as the control feed substrate) with gradual addition of various proportions of the powdered tomato plant biomass (Figure 2). Each formulation consisted of poultry feed pellets, mixed with an amount of dried tomato plant biomass. The latter was incorporated with respect to the following inclusion levels: 0% (control), 10%, 20%, 40%, 60%, 80%, and 100%, all calculated on a dry weight basis (*w*/*w*). To each formulation, an amount of tap water was added to adjust their moisture level uniformly to 70%, which constitutes the optimal condition for larvae growth. Then, a homogenization step was implemented to ensure that the disintegrated pellets and the dry tomato plant biomass were thoroughly homogenized and these components were evenly distributed. Thus, a total of seven dietary treatments were created, with respect to the tomato biomass amount incorporated into poultry feed. All experimental diets were freshly prepared before each feeding event to minimize nutrient oxidation or degradation.

### 3.4. BSF Larvae Rearing

**Figure 3:**
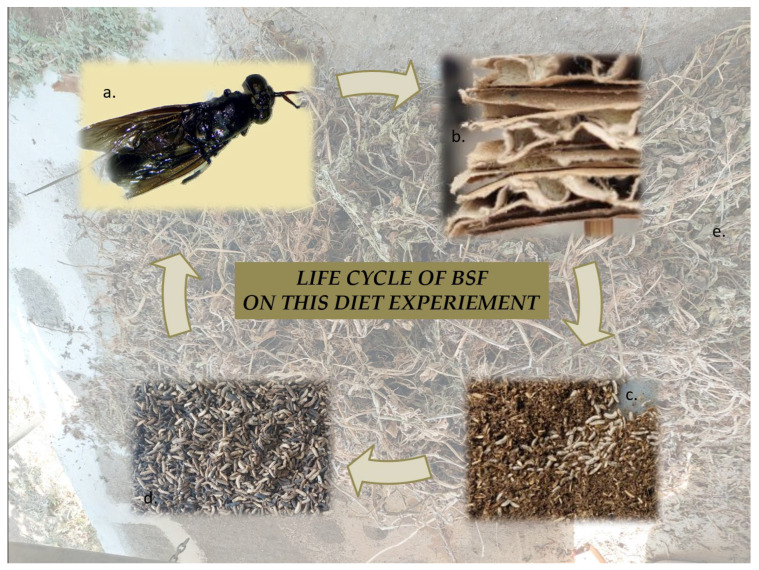
BSF life cycle: (**a**) BSF adult, (**b**) BSF eggs laying between the papers’ holes, (**c**) 1st to 4th instar stage of BSF larvae, (**d**) 6th instar stage of BSF larvae, and (**e**) potato plant dried biomass.

BSF eggs were provided by the Laboratory of Agricultural Zoology and Entomology of the Agricultural University of Athens, from its permanently maintained BSF colony. The eggs were incubated until hatching. On the 5th day, 200 of the newly emerged larvae were transferred into separate rearing containers of 14.5 × 9.5 × 5 cm size. Thus, a fixed density per container was maintained and a consistency across all treatments was ensured. Rearing was implemented in these growth chambers in darkness, at 30 °C controlled temperature and humidity of 70%, to achieve an uninterrupted development of larvae. The larvae were fed every three days with 48 g of the assigned experimental diet per container. Prior to each feeding event, the larvae were rinsed with deionized water and gently dried, and a subsample of 30 individuals was separated, and their body weights and lengths were measured to monitor growth progression. Rearing was continued until larvae reached the 6th instar stage (approximately the 16th day), which was set as their sampling time (Figure 3). The latter constitutes the instar stage commonly used for BSF larvae by feed industry. The mortality rate for all treatments was below 2%, a value acceptable for their rearing. At harvest, all experimental larvae were collected, washed, frozen at −20 °C, lyophilized, ground to fine powder, and stored under dry, dark conditions until their analyses.

### 3.5. Assessment of Nutritional Indicators

The nutritional composition of the produced insect meals and tomato plant biomass were determined by applying standard analytical methodologies. Specifically, the total protein content was quantified using the Kjeldahl method in accordance with the official AOAC method [40]. The ash content was determined for dry aching in a muffle furnace at 550 °C following the respective AOAC guideline [40]. Crude fiber content was measured using an automated fiber analysis system, while the contained structural fiber components (lignin and cellulose) were assessed using the acid-detergent fiber (ADF) procedure [41]. For the determination of the total lipid content, samples were extracted in triplicate with hexane under continuous stirring, and the combined extracts were evaporated to dryness under reduced pressure using a rotary evaporator of laboratory scale. All determinations were performed in triplicate and respective results have been expressed as mean ± standard deviation.

### 3.6. Phenolic Content and Antioxidant Capacity Evaluation

The Total Phenolic Content (TPC) was determined by applying a previously reported spectrophotometric method [42]. In specific, triplicate samples were placed into a 96-well microplate (Sarstedt AG & Co. KG, Nümbrecht, Germany) and the respective absorbances were measured in a NanoQuant, infinite M200PRO (Tecan Group Ltd., Männedorf, Switzerland) instrument. The TPC was determined at the 765 nm absorption wavelength, and the respective value was calculated using a gallic acid standard calibration curve. The results are expressed as mg of gallic acid equivalents per g of extract (mg GAE/g extract).

The antioxidant capacities of investigated samples were estimated by performing the 2,2-DiPhenyl-1-PicrylHydrazyl (DPPH^•^) and the Ferric Reducing Antioxidant Power (FRAP) assays. The determinations were conducted in triplicate, and the respective results have been expressed as mean values ± standard deviation. Absorbance measurements were carried out using an Infinite^®^ 200 PRO microplate reader (Tecan Group Ltd., San Jose, CA, USA). Both assays were performed on the methanolic extracts of the insect meals produced from each experimental diet. For this purpose, 200 mg of each insect meal sample was poured into 1 mL of methanol and stirred at room temperature for 20 min. Then, the supernatant was separated and used for the determination of its antioxidant capacity.

For the antioxidant ability estimation with the DPPH^•^ assay, 30 µL of each sample was mixed with 175 µL of DPPH solution and the mixture incubated for 40 min at room temperature in the absence of light. Then, the absorbance was measured at 515 nm, and the antioxidant activity was calculated using a Trolox standard curve, providing the respective results expressed as mg Trolox equivalents (TE)/kg of dry material (y = −0.0060215x + 0.82922, r = 0.99941).

The antioxidant potential was also estimated with the FRAP assay that measures the samples’ ability to reduce ferric (Fe^3+^) to ferrous iron (Fe^2+^). Briefly, 30 µL of each sample was combined with 180 µL of freshly prepared FRAP reagent in a 96-well microplate and incubated at 37 °C for 30 min. Subsequently, the absorbance was recorded at 593 nm, and the samples’ quantification was achieved using a calibration curve, providing the respective results expressed as mol Fe^2+^/kg of dry matter (y = 0.59678x + 0.097217, r = 0.99962).

### 3.7. Determination of Toxic Alkaloid Content with UHPLC-HRMS Analysis

The presence of toxic alkaloids was determined in the insect meal obtained from the BSF feeding ratio highlighted herein as the most efficient (40% incorporation of tomato plant biomass). Additionally, for comparison purposes, we have also determined the respective values for insect meals composed by poultry feed (0%) and tomato plant biomass (100%). The analyses were performed using a Dionex Ultimate 3000 UHPLC system (Thermo Fisher Scientific, San Jose, CA, USA) coupled to a Q-Exactive Orbitrap HRMS (Thermo Fisher Scientific, San Jose, CA, USA). The chromatographic separation was achieved using a Hypersil Gold UPLC C18 (2.1 mm × 150 mm, 3 μm) reverse-phased column (Thermo Fisher Scientific, San Jose, CA, USA). The mobile phases consisted of water (A) and methanol (B), both containing 0.1% formic acid and 10 mM ammonium formate, in accordance with the following gradient program: 0–2 min 5% B, 2–20 min from 5% to 95% B, 20–23 min 95% B, 23–24 min from 95% to 5% B, and 24–30 min 5% B. The column temperature was set at 40 °C, the flow rate was 0.3 mLmin-1, and the injection volume was 10 μL. Mass spectrometric detection was performed in positive ion mode, with the following parameters: capillary temperature 350 °C, sheath gas 40 units, auxiliary gas flow 5 units, spray voltage 2.7 kV, and S-lens 50 V. Data acquisition was performed in full scan mode in the range 80–1200 *m*/*z*, with a resolution of 70,000, complemented by data-dependent MS/MS acquisition for structural elucidation of the most intense ions using stepped collision energies (20, 40, and 60), with a resolution of 17,500. Data acquisition and processing were carried out using the Xcalibur 4.0 software (Thermo Fisher Scientific, San Jose, CA, USA).

### 3.8. Statistical Analysis

All analyses were performed in triplicate, and the respective results have been expressed as means ± standard deviation (±S.D.). The normality of the data distribution was evaluated using the Kolmogorov–Smirnov and Shapiro–Wilk tests. The examined parameters were compared across treatments by applying one-way ANOVA (Statgraphics centurion v.19) for parametric data after Tukey’s adjustment for multiplicity, while Kruskal–Wallis analysis was performed for nonparametric data.

Prior to analysis, the results of toxic alkaloid response variable were log-transformed to better satisfy ANOVA assumptions. After transformation, the two-way ANOVA indicated a significant overall model (F = 38.89, *p* = 0.0001), explaining a high proportion of variability (R^2^ = 0.978; adjusted R^2^ = 0.953).

## 4. Conclusions

This study constitutes a pioneering framework for the circular valorization of tomato plant (*Solanum lycopersicum*) biomass, by transforming a previously underutilized and potentially hazardous agricultural waste into a high-value feed ingredient. Although several agro-industrial byproducts are currently in use for BSF rearing [43,44,45], the epitome of this research lies in the groundbreaking demonstration that BSF larvae may function as bio-convertors for the efficient detoxification of *Solanaceae* toxic cultivation residues. For the first time, it is evidenced that this insect-mediated process can drastically reduce the concentration of toxic steroidal alkaloids, achieving an attenuation of α-tomatine levels by exceeding 90%. This finding is of paramount importance as it successfully overcomes the primary obstacle of toxicity that prohibits the utilization of tomato cultivation residues in animal nutrition, thereby creating safe, sustainable, and abundant raw materials that enhance the resilience of food production systems.

Among the dietary formulations evaluated, the 40% inclusion level of tomato biomass emerged as the optimal choice, representing a balanced waste utilization and high-quality nutritional output. At this specific ratio, the resulting insect meal reached its peak protein concentration (30.61%), while a robust lipid profile was maintained with the presence of a controlled fiber content and a significantly enhanced antioxidant capacity. The incorporation of 40% tomato biomass into BSF-feeding rations ensures the optimal larvae growth and metabolic efficiency, avoiding the physiological constraints observed for higher substitution rates. This formulation was highlighted as preferrable since the 60% formulation provided insect meal of lower nutritional content, while the 20% formulation that yielded insect meal of comparable nutritional value valorized a substantially lower amount of tomato plant biomass.

The results herein revealed that the strategic integration of tomato cultivation residues into BSF diets not only neutralizes the naturally occurring plant toxins but also fulfills the escalating demand for nutrient-dense functional feeds through the effective conversion of an environmental waste challenge into a strategic opportunity for a profitable and sustainable form of livestock nutrition [46].

However, certain limitations of the present study should be acknowledged. The experimental design was conducted under controlled laboratory conditions, which may not fully reflect large-scale industrial rearing environments. In addition, the study focused on a single tomato biomass source and did not account for the potential variability in the phytochemical composition arising from different cultivars and cultivation practices. Moreover, while the reduction in toxic alkaloids was demonstrated, the underlying metabolic pathways involved in their degradation by BSF larvae were not elucidated.

Future research should therefore aim to address these limitations by investigating the scalability and economic feasibility of this bioconversion process under industrial conditions, as well as assessing the consistency of results across diverse tomato biomass sources. Further studies should also focus on elucidating the biochemical and microbiological mechanisms responsible for alkaloid degradation within the larvae. In addition, long-term feeding trials in livestock are required to validate the safety, digestibility, and functional performance of the produced insect meal.

## Figures and Tables

**Table 1:** Growth performance metrics (weight, length, and weight-to-length ratio) of BSF larvae reared with varying levels of tomato biomass (weight *p* < 0.001; length *p* < 0.05; weight/length *p* < 0.05).

	BSF Diets						
	T0%	T10%	T20%	T40%	T60%	T80%	T100%
Weight (mg)	239.47 ± 49.6 ^c^	235.76 ± 34.68 ^c^	194.80 ± 24.33 ^b^	193.91 ± 33.28 ^b^	203.61 ± 30.88 ^b^	187.50 ± 23.32 ^b^	116.60 ± 25.48 ^a^
Length (mm)	21.17 ± 1.94 ^c^	23.97 ± 1.34 ^d^	23.23 ± 1.73 ^d^	21.12 ± 3.1 ^c^	21.24 ± 2.11 ^c^	19.49 ± 1.34 ^b^	14.83 ± 1.50 ^a^
Weight (mg)/Length (mm)	11.31 ± 0.12 ^d^	9.84 ± 0.16 ^c^	8.39 ± 0.15 ^b^	9.18 ± 0.71 ^bc^	9.59 ± 0.17 ^c^	9.62 ± 0.28 ^c^	7.86 ± 0.12 ^a^

For each line, the exponents a, b, c, and d indicate the respective grouping (a < b < c < d).

**Table 2:** Nutritional composition, fiber fractions, and mineral content of Tomato Plant Biomass and Poultry Feed used as row materials for the preparation of BSF feed.

		Tomato Plant Biomass	Poultry Feed
Proteins%		14.05 ± 1.65	15.00 ± 0.89
Sugars%		1.56 ± 0.26	3.26 ± 0.53
Crude Fiber Substances	Total%	40.74 ± 1.17	3.80 ± 0.44
	Lignin%	8.83 ± 0.58	1.20 ± 0.20
	Cellulose%	37.95 ± 1.64	2.60 ± 0.38
Total Fats%		1.27 ± 0.34	3.20 ± 0.65
Ash%		19.65 ± 3.64	6.00 ± 0.71

**Table 3:** Nutritional composition, fiber fractions, and mineral content of insect meals derived from BSF larvae rearing with different tomato plant inclusion levels (proteins *p* < 0.001; sugars *p* > 0.05; crude fiber substances—total *p* < 0.001; crude fiber substances—lignin *p* < 0.01; crude fiber substances—cellulose *p* < 0.05; total fat *p* < 0.001; and ash *p* < 0.001).

		BSF-Derived Insect Meals						
		T0%	T10%	T20%	T40%	T60%	T80%	T100%
Proteins%		20.58 ± 2.86 ^a^	21.44 ± 1.94 ^a^	27.20 ± 3.98 ^b^	30.61 ± 0.65 ^c^	29.04 ± 1.16 ^bc^	29.72 ± 1.59 ^bc^	18.39 ± 14.27 ^a^
Sugars%		1.53 ± 0.48	2.01 ± 0.08	2.56 ± 0.87	1.93 ± 0.54	2.57 ± 0.42	1.68 ± 0.47	2.28 ± 0.65
Crude Fiber Substances	Total%	6.45 ± 1.25 ^ab^	6.95 ± 0.87 ^ab^	7.89 ± 0.27 ^b^	6.45 ± 1.01 ^ab^	6.27 ± 0.84 ^a^	7.42 ± 0.89 ^ab^	11.87 ± 0.96 ^c^
	Lignin%	1.82 ± 0.51 ^a^	1.95 ± 0.46 ^a^	1.76 ± 0.34 ^a^	2.47 ± 0.93 ^ab^	2.58 ± 0.42 ^ab^	3.07 ± 0.49 ^b^	3.29 ± 0.45 ^b^
	Cellulose%	13.40 ± 0.68 ^ab^	14.02 ± 1.24 ^abc^	15.74 ± 1.71 ^c^	15.31 ± 0.65 ^c^	12.38 ± 0.97 ^a^	14.88 ± 1.70 ^bc^	13.09 ± 1.09 ^ab^
Total Fats%		29.82 ± 1.56 ^d^	30.59 ± 2.46 ^d^	28.63 ± 0.96 ^d^	29.54 ± 1.67 ^d^	24.73 ± 2.64 ^c^	18.61 ± 1.84 ^b^	6.62 ± 0.98 ^a^
Ash%		10.62 ± 0.41 ^ab^	10.48 ± 1.21 ^ab^	9.97 ± 0.31 ^a^	11.05 ± 0.29 ^ab^	12.41 ± 0.98 ^bc^	13.87 ± 1.72 ^c^	23.18 ± 2.59 ^d^

For each line, the exponents a, b, c, and d indicate the respective grouping (a < b < c < d).

**Table 4:** Total phenolic yield and antioxidant capacity (DPPH and FRAP assays) of BSF-derived meals across experimental treatments (total phenolic content *p* < 0.001; antioxidant capacity evaluation—DPPH *p* < 0.001; antioxidant capacity evaluation—FRAP *p* > 0.05).

		BSF-Derived Insect Meals						
		T0%	T10%	T20%	T40%	T60%	T80%	T100%
Total Phenolic Content (mg/mL)		17.38 ± 0.94 ^b^	16.72 ± 1.26 ^b^	19.83 ± 4.19 ^b^	19.96 ± 3.72 ^b^	18.59 ± 3.94 ^b^	11.47 ± 4.54 ^a^	8.74 ± 1.39 ^a^
Antioxidant Capacity Evaluation	DPPH (mg/mL)	17.29 ± 6.59 ^c^	27.11 ± 2.54 ^d^	22.67 ± 2.09 ^cd^	22.56 ± 3.01 ^cd^	21.14 ± 3.04 ^c^	11.18 ± 4.09 ^b^	1.40 ± 0.88 ^a^
	FRAP (mg/mL)	0.56 ± 0.36	0.61 ± 0.06	0.59 ± 0.38	0.69 ± 0.32	0.63 ± 0.13	0.53 ± 0.21	0.32 ± 0.08

For each line, the exponents a, b, c, and d indicate the respective grouping (a < b < c < d).

**Table 5:** Concentrations of steroidal alkaloids expressed as ppm of α-tomatine and tomatidine in tomato plant substrate and the produced BSF-*larvae*-rearing meals.

Insect Meal	α-Τomatine	Tomatidine
T0%	<LOQ	<LOQ
T40%	109.637 ± 16.629	26.678 ± 3.409
T100%	216.819 ± 15.230	34.607 ± 3.104
Plant Biomass	1166.917 ± 14.689	70.591 ± 11.387

LOQ: Limit of Quantitation.

## Data Availability

The original contributions presented in this study are included in the article. Further inquiries can be directed to the corresponding author.
